# Recent Advances in Polyoxometalates Targeting Proteins Associated with Alzheimer’s Disease: From Molecular Mechanisms to Therapeutic Applications

**DOI:** 10.3390/ijms27031257

**Published:** 2026-01-27

**Authors:** Lijuan Zhang, Tinghao Lu, Ziqian Hua, Shiheng Peng, Haoming Du, Xiaoting Zhai, Zhiqiang Cai, Jiai Hua, Xiang Ma

**Affiliations:** 1 Chemistry and Chemical Engineering Department, Taiyuan Institute of Technology, Taiyuan 030008, China; 16634218541@163.com (L.Z.); lutinghao61@163.com (T.L.); huaziqian06@163.com (Z.H.); duhaoming00@163.com (H.D.); zhaixiaoting111@163.com (X.Z.); 2 School of Petrochemical Engineering, Shenyang University of Technology, Liaoyang 111000, China; kahongzqc@163.com; 3 Laboratory of Biochemistry and Pharmacy, Taiyuan Institute of Technology, Taiyuan 030008, China; pengshihengpsh@163.com

**Keywords:** polyoxometalates, Alzheimer’s disease, protein misfolding, nanomedicine, enzyme inhibition, photodynamic therapy

## Abstract

Polyoxometalates (POMs) exhibit significant potential for application in Alzheimer’s disease (AD) therapeutics owing to their inherent chemical and physical properties and structural tunability. Through transition metal substitution, functional modification, and the construction of POMs-based nanocomposites, POMs can precisely recognize and effectively modulate various key pathogenic proteins involved in Alzheimer’s disease. They can also intervene in disease progression through multiple mechanisms, including inhibition of Aβ aggregation, disaggregation of amyloid-β (Aβ), scavenging of reactive oxygen species (ROS), hydrolytic activity, and modulation of enzyme function. In addition, due to their outstanding physicochemical properties, the application of POMs in phototherapy has emerged as a significant direction in AD treatment research. This review systematically summarizes recent advances from 2011 to 2025 in POMs targeting key pathogenic proteins in AD, comprehensively analyzes their specific mechanisms of action across different therapeutic contexts, highlights their significant advantages and broad potential in AD treatment, and provides new insights for the future structural design, functional optimization, and clinical translation of POMs.

## 1. Introduction

Alzheimer’s disease (AD) is a neurodegenerative disorder of the central nervous system characterized by progressive cognitive decline, with complex and multifaceted pathogenic mechanisms [1]. Based on studies of AD pathogenesis, ten core protein targets associated with the disease have been identified to date. These include β-secretase 1 (BACE1), presenilin 1 (PSEN1), presenilin 2 (PSEN2), amyloid precursor protein (APP), and apolipoprotein E (APOE), which are involved in amyloid-β (Aβ) generation and Aβ aggregation; microtubule-associated protein tau (MAPT), related to Tau pathology; α-synuclein (SNCA), implicated in synaptic function; acetylcholinesterase (AChE) and butyrylcholinesterase (BChE), which regulate the cholinergic system; and brain-derived neurotrophic factor (BDNF), associated with neuroprotection [2]. The design of current therapeutic strategies for AD is primarily centered on the pathological mechanisms or protective functions associated with the aforementioned targets [3,4,5]. Although extensive research has been conducted on AD treatment, no drugs have been developed that can effectively cure the disease, and the development of novel therapeutic agents remains urgently needed [6].

Polyoxometalates (POMs) are discrete metal-oxygen clusters composed of early transition metals (such as V, Mo, W, and Nb) in their highest oxidation states, and their structural tunability and multifunctionality arise from the ability to incorporate a wide range of elements from the periodic table into their frameworks [7]. This property endows POMs with outstanding physicochemical properties and enables them to specifically bind to proteins through diverse intermolecular forces, thus conferring significant biological activity [8]. In addition to their chemical versatility, polyoxometalates exhibit remarkable modularity and structural programmability at the molecular level. For example, polyoxoniobates have been used as molecular building blocks to construct thin films, showing that even clusters with similar inorganic cores can be assembled in highly controllable ways with diverse functions. Such studies emphasize that POM properties strongly depend on precise structural design, providing a conceptual basis for rational modification to tailor their interactions with biological targets, including proteins relevant to Alzheimer’s disease [9]. From a physicochemical perspective, thermodynamic studies provide fundamental insights into polyoxometalate–biomolecule recognition. For instance, the interaction between the Keplerate-type polyoxometalate {Mo_72_Fe_30_} and vitamin B1 has been shown to be spontaneous, driven by a combination of electrostatic interactions, hydrogen bonding, and van der Waals forces, thereby providing fundamental physicochemical evidence for polyoxometalate–biomolecule recognition [10]. POMs possess a high density of negatively charged surface sites, enabling electrostatic interactions with positively charged regions of proteins, such as those containing ammonium groups in lysine residues and guanidinium groups in arginine residues [11,12]. In addition, POMs can form hydrogen bonds directly with polar uncharged amino acid residues—such as tyrosine, serine, and asparagine—on protein surfaces in aqueous or metal ion-containing media [11,13]. The metal centers in transition metal-substituted POMs can form coordination bonds with oxygen or nitrogen atoms in proteins—such as those present in histidine side chains and in peptide backbones [14,15,16,17]—whereas POMs covalently modified by organic ligands are capable of engaging in additional hydrophobic interactions with hydrophobic regions of proteins through the ligands [18,19,20,21]. Notably, the size of POMs, their charge density, and the solution environment, including pH, ionic strength, and temperature, can dynamically modulate the strength of their interactions with proteins [22]. When the charge density of POMs is at a moderate level, it not only maintains a strong electrostatic attraction to proteins but also balances the desolvation energy, resulting in optimal binding affinity [23,24]. In addition, the greater the structural complementarity between POMs and proteins in terms of size and shape, the stronger the interaction between them [23,25]. Regarding the effect of solution conditions on the electrostatic interactions between POMs and proteins, specifically, the electrostatic attraction decreases with increasing ionic strength of the solution [26]. The pH, on the other hand, influences the interaction strength between POMs and proteins by altering the net charge on the protein surface [27]. The effect of temperature on the electrostatic interactions between POMs and proteins is more complex than that of ionic strength and pH [28,29,30]. The core pathologies of AD—abnormal Aβ deposition and hyperphosphorylation of tau proteins—are both closely associated with protein misfolding, aggregation, and intermolecular interactions [31]. Therefore, the elemental composition, charge distribution, and spatial configuration of POMs can be deliberately tailored to enable specific recognition and interaction with key AD target proteins, thereby interfering with the aggregation process of pathological proteins, blocking the neurotoxic cascade, and offering a novel therapeutic strategy for AD treatment.

Early studies have shown that although purely inorganic POMs can bind to the positively charged regions of amyloid-beta peptides via electrostatic interactions to inhibit their aggregation [32], their high toxicity and long-term safety concerns have limited clinical applicability [33]. Currently, research on POMs is no longer limited to purely inorganic POMs but has shifted toward functionally tailored designs [34]. Among the functionalization strategies, transition metal-substituted POMs can enhance binding affinity by forming coordination bonds with the histidine and cysteine residues of Aβ [15]. Additionally, modification with chiral ligands enables enantioselective inhibition of Aβ aggregation [35,36]. Targeting peptide modification can effectively reduce the neurotoxicity of Aβ through specific recognition of Aβ by targeting peptides [37]. In addition, the construction of POM-based nanocomposites can not only enable integrated multicomponent synergistic therapy but also further enhance blood–brain barrier penetration efficiency and in vivo metabolic safety [38].

Notably, the pathological progression of AD involves multiple interconnected and mutually reinforcing cascades, each closely linked to specific pathogenic proteins [39]. Among these cascades, Aβ aggregation may act as the initiating event of the pathological process, triggering downstream pathological events such as tau protein hyperphosphorylation and neuroinflammation [40]. Meanwhile, cholinergic system dysfunction, mediated by cholinesterases, serves as a key driver of cognitive decline, as these enzymes accelerate the degradation of acetylcholine, thereby exacerbating cognitive impairment [41]. While neuroinflammation mediated by the pro-inflammatory calcium-binding protein S100 calcium-binding protein A9 (S100A9) acts as an amplifier of the pathological process by promoting Aβ self-assembly and exacerbating neuronal damage, it further propels disease progression [42]. Given the central roles of these proteins in the pathogenesis of AD, they may serve as prime targets for multidimensional therapeutic interventions. Among the ten core AD-related proteins, BACE1, PSEN1, PSEN2, APP, APOE, and MAPT are not discussed in detail in this review because studies on their interactions with POMs remain limited or at an early stage. In contrast, substantial experimental and computational evidence supports the binding and modulatory effects of POMs on Aβ aggregation, as well as inhibitory interactions with cholinesterases (AChE and BChE) and the pro-inflammatory amyloidogenic protein S100A9. This review focuses on the latest research advances in POMs targeting these four key proteins, systematically elucidates the specific mechanisms of POM-protein interactions, such as electrostatic, coordination, and hydrophobic interactions, and analyzes how structural features of POMs, including charge density, molecular size, and functional modifications, regulate their biological activities, such as inhibiting Aβ aggregation, modulating cholinesterase activity, and mitigating neuroinflammation.

## 2. POMs in the Inhibition of Aβ Aggregation

### 2.1. Inhibition of Aβ Aggregation by Pure POMs

Purely inorganic POMs serve as the fundamental systems for POM-based inhibition of Aβ aggregation [43]. Their inhibitory performance is mainly determined by intrinsic structural parameters, such as molecular size and surface charge density, and they modulate Aβ aggregation through electrostatic and other intermolecular interactions. These characteristics provide fundamental structural references for subsequent functional modifications [44]. Owing to their high structural versatility, tunable physicochemical properties, and rich coordination chemistry, POMs have considerable potential for biomedical applications [45]. In 2011, the Qu group demonstrated for the first time that a series of POMs can inhibit the aggregation of amyloid-β peptides [32]. Among these POMs, the Wells-Dawson structures exhibited the strongest inhibitory activity, Keggin-type POMs showed moderate to high activity, whereas Anderson-type structures displayed negligible inhibition. These results suggest that molecular size is a critical determinant of POM-Aβ recognition and inhibitory efficacy, while surface charge contributes an essential complementary influence. Highly negatively charged POMs preferentially interact with positively charged residues—such as Lys and Arg—on the Aβ surface, with especially strong affinity for the cationic His13–Lys16 (HHQK) cluster. This initial structure-activity relationship study not only validated the potential of purely inorganic POMs as inhibitors of Aβ aggregation, but also delineated the key structural parameters—namely molecular size and surface charge—that govern their activity. These insights laid an essential experimental foundation for the rational design of next-generation POM-based materials targeting Alzheimer’s disease. Table 1 summarizes the applications of POMs in Alzheimer’s disease therapy.

In 2014, Chen et al. designed spherical pure inorganic POM nanoclusters based on {Mo_11_} fragments, further expanding the functional scope of pure inorganic POMs [47]. These POM nanoclusters exhibit a high negative charge density together with a rigid, cage-like framework. They inhibit Aβ_40_ self-aggregation through electrostatic interactions and additionally suppress metal-ion-induced Aβ aggregation by occupying Zn^2+^/Cu^2+^ binding sites and chelating unbound metal ions. In contrast to conventional organic chelators, these purely inorganic POMs function without ligand-specific coordination. Rather, their intrinsic structural features enable both inhibition of Aβ self-aggregation and suppression of metal-induced aggregation. Moreover, these nanoclusters display substantial stability under physiological pH conditions, providing a viable strategy for mitigating metal-ion dyshomeostasis associated with AD pathology.

**Figure 1:**
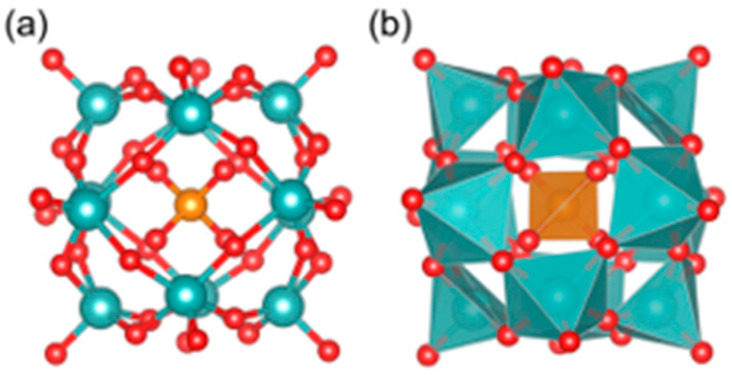
The ball-stick model (**a**) and polyhedral model (**b**) of [PO_40_Mo_12_]^3−^. Red color is used for oxygen atoms, dark orange for phosphorus, and cyan blue for molybdenum [48].

Recently, Fang et al. investigated the pure inorganic POM [PO_40_Mo_12_]^3–^ (Figure 1) [48]. This work employed, for the first time, a multiscale computational framework that integrates active-learning Bayesian optimization (BO) with density functional theory (DFT) to demonstrate at the atomic level that [PO_40_Mo_12_]^3–^ binding is not dominated by strong electrostatic interactions. Instead, it achieves binding through the formation of multivalent weak interactions (e.g., hydrogen bonds and van der Waals forces) between its surface oxygen atoms and the side chains of Aβ amino acids. This interaction mode minimizes the structural perturbation typically associated with strong electrostatic forces and simultaneously impedes Aβ self-assembly through steric effects. Moreover, the intrinsic −3e charge produces moderate Coulombic repulsion that balances the overall binding strength and contributes to biocompatibility. The large Highest Occupied Molecular Orbital (HOMO)-Lowest Unoccupied Molecular Orbital (LUMO) gap further helps preserve the structural integrity of Aβ. Collectively, these findings offer molecular-level insights that can guide the rational design of low-toxicity POMs.

### 2.2. Transition Metal Substituted POMs

Transition-metal substitution is an important strategy for enhancing the interactions between POMs and Aβ [64]. Incorporation of transition-metal ions (e.g., Ni^2+^ and Co^2+^) enables modulation of the POM surface charge density and enhances specific Aβ recognition through metal–ligand coordination, thereby shifting the mechanism from non-specific inhibition to more precise molecular targeting [65].

**Figure 2:**
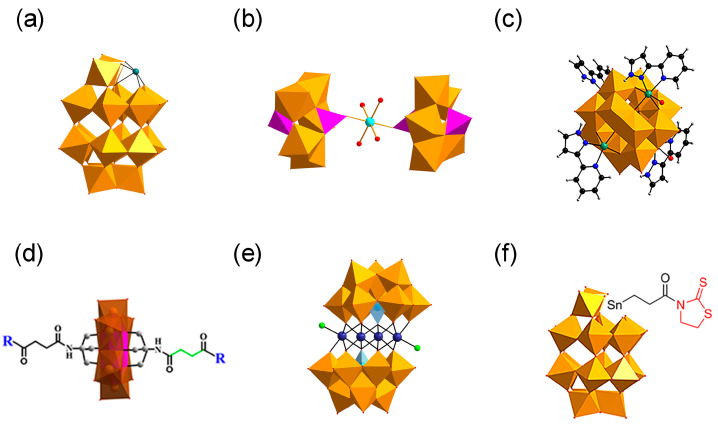
Structures of polyoxometalates (POMs). (**a**) Wells–Dawson POMs with defined histidine-chelating metals. (**b**) K_8_{[Co(H_2_O)_4_][HP_2_Mo_5_O_23_]_2_}·8H_2_O. (**c**) {[CoL-(H_2_O)]_2_[CoL]_2_[HAs^V^Mo^VI^_6_O_40_]} (CAM). (**d**) D/L-amino acid-functionalized POM derivatives. (**e**) (H_2_dap)_6_[CdCl_2_(B-α-AsW_9_O_34_)_2_]8·H_2_O. (**f**) TZ-modified POM derivative (POMD-TZ), the Wells–Dawson POM covalently modified with thiazolidinethione (TZ) through a Sn-propionyl linker (Sn-CH_2_CH_2_C(O)-TZ).

**Figure 3:**
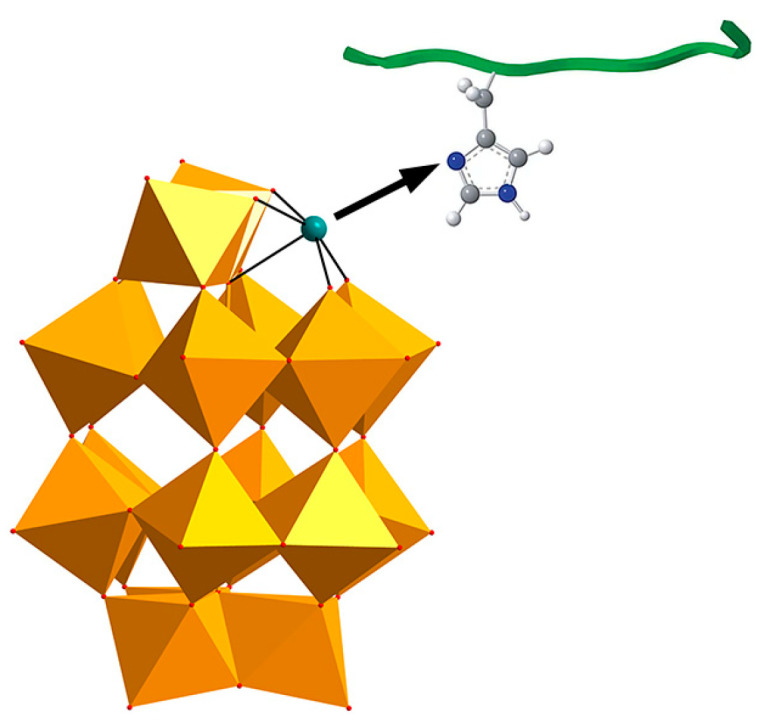
The chelation between the transition metals in POM and the histidine in amyloid-β (Aβ) monomer. The WO_6_ polyhedra is shown in gold. The histidine-chelating metal is shown as a teal ball. The O, C, N and H atoms are shown as red, grey, dark blue and white balls, respectively [15].

Building upon the earlier structure–activity relationship findings that highlighted molecular size and surface charge as key determinants for the anti-Aβ activity of pure inorganic POMs, Qu and co-workers further advanced this framework in 2014 by introducing transition-metal substitution into Wells-Dawson-type POMs and systematically evaluating their inhibitory effects on Aβ aggregation (Figure 2a) [15]. Metal substitution increases the surface negative charge density of POMs, significantly strengthening electrostatic interactions with the HHQK cationic cluster of Aβ. In addition, Ni^2+^ and Co^2+^ can coordinate with the imidazole nitrogen atoms of adjacent histidine residues (Figure 3), providing additional binding specificity and reinforcing the overall interaction with Aβ. More importantly, this class of POMs can block the coordination between Aβ and heme (with His13/His14 serving as key binding sites for the Aβ-heme complex), thereby significantly inhibiting the peroxidase activity of the Aβ-heme complex and reducing reactive oxygen species (ROS) production. In vivo studies further showed that POMds-Dawson-Ni is capable of crossing the blood–brain barrier, is metabolized within 48 h, and exhibits no detectable toxicity, suggesting its potential as a bifunctional agent that inhibits Aβ aggregation and mitigates oxidative stress. 

In 2023, our group synthesized a cobalt complex-functionalized Strandberg-type phosphomolybdate, K_8_{[Co(H_2_O)_4_][HP_2_Mo_5_O_23_]_2_}·8H_2_O (abbreviated as CoPM) (Figure 2b) [49]. Structurally, CoPM consists of two Strandberg-type [P_2_Mo_5_O_23_]^6−^ units bridged by a [Co(H_2_O)_4_]^2+^ complex, forming a nanoscale sandwich-like architecture. This structural design endows CoPM with the synergistic advantages of spatial matching, electrostatic binding, and coordination targeting. Specifically, its nanoscale dimensions allow steric interference with β-sheet formation; its oxygen-rich surface interacts with cationic Aβ residues through electrostatic and hydrogen-bonding interactions; and the central Co^2+^ enhances molecular targeting through coordination with histidine imidazole groups. As a result, CoPM inhibits Aβ fibrillation and reduces ROS production from Cu^2+^-Aβ complexes, enabling simultaneous intervention in two pathological pathways relevant to Alzheimer’s disease.

In summary, incorporating transition-metal centers enhances the targeted binding of POMs to Aβ by increasing surface charge density and enabling metal-histidine coordination, while also improving spatial complementarity through metal-mediated structural regulation. In addition, these substitutions confer further functional benefits, including attenuation of Aβ-heme activity and reduction in ROS levels. 

### 2.3. Inorganic-Organic Hybrid POMs

In recent years, organic–inorganic hybrid polyoxometalates have attracted considerable attention not only in the field of catalysis, while their potential relevance in bio-related research has also gradually emerged. Studies indicate that the incorporation of organic components can effectively modulate the interactions between POM-based materials and biological systems, thereby highlighting that their biological effects are highly dependent on molecular structural design [66]. In the context of Alzheimer’s disease, inorganic-organic hybrid POMs constructed via organic ligand modification, chiral component incorporation, or functional group grafting integrate the structural stability of inorganic POMs with the functional diversity of organic components [67]. This integration specifically mitigates the lack of specificity in the interaction between pure inorganic POMs and Aβ [68]. Such materials enable precise regulation of the Aβ aggregation process via hydrophobic interactions, hydrogen bonding, or chiral recognition between their organic moieties and Aβ, while retaining the capacity of the inorganic framework to interfere with Aβ conformational transitions. This offers a more flexible structural design strategy for Aβ-targeted intervention in AD therapy.

**Figure 4:**
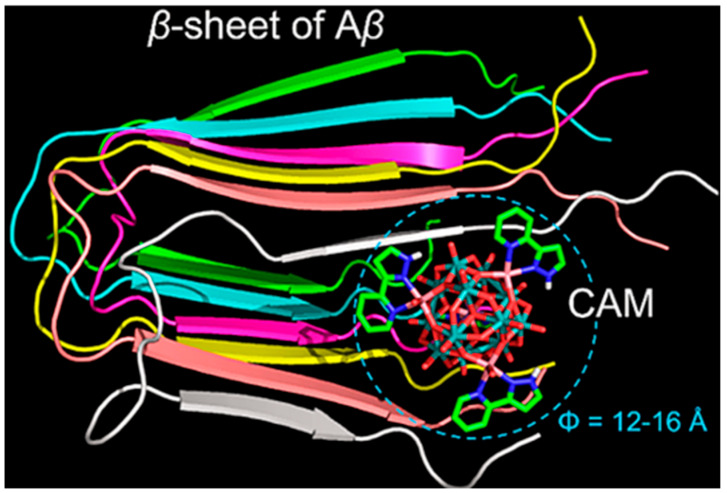
Overview of the CAM–Aβ complex represented in a space filling mode. The β-sheet strands of Aβ are colored differently to distinguish individual peptides. In the CAM, oxygen atoms are colored red, nitrogen atoms blue, carbon atoms green, cobalt atoms pink, and molybdenum atoms cyan [50].

To achieve precise targeted intervention in Aβ aggregation, in 2018, our team designed and synthesized an ε-Keggin-type POM, {[CoL-(H_2_O)]_2_[CoL]_2_[HAs^V^Mo^VI^_6_O_40_]} (abbreviated as CAM) (Figure 2c), which is the first reported β-sheet conformation modifier directly targeting Aβ for AD therapy [50]. The surface oxygen atoms of CAM form hydrogen bonds with specific amino acid residues in the cavity of Aβ β-sheet aggregates. Its L group serves as a β-sheet targeting moiety and effectively recognizes the β-sheet conformation of Aβ. These two functionalities significantly enhance the targeting specificity of CAM toward Aβ aggregates. The overall dimensions of CAM match the cavity of β-sheet aggregates, allowing it to stably embed within this space (Figure 4). By perturbing the intermolecular hydrogen-bond network of β-sheet aggregates, CAM induces a transition of Aβ from a β-sheet to a non-β-sheet conformation, thereby inhibiting the formation of toxic aggregates and promoting the disassembly of preformed Aβ aggregates. Additionally, the lipophilicity and near-electroneutral character of CAM facilitate its penetration across the bloodbrain barrie (BBB) and enable its binding to brain Aβ aggregates, thereby exerting therapeutic effects in vivo relevant to Alzheimer’s disease.

Chirality is widespread in nature, and the basic building blocks of living organisms are chiral (e.g., amino acids, sugars, proteins, and DNA) [69,70]. Due to their strong negative charge and nanoscale dimensions, POMs readily engage in non-specific electrostatic interactions with biomolecules, thereby exhibiting broad application potential in the biomedical field [71]. However, the interaction of POMs with biomolecules mainly relies on non-specific forces (e.g., electrostatic attraction) and lacks chiral recognition capability [72]. As a polypeptide containing multiple chiral carbon atoms, Aβ possesses inherent chiral characteristics [73]. When aggregated into protofibrils, its secondary structure transitions from an α-helix to β-sheet with a complex chiral architecture [74], which further reinforces the chiral cascade and confers specific sensitivity to chiral environments [75].

**Figure 5:**
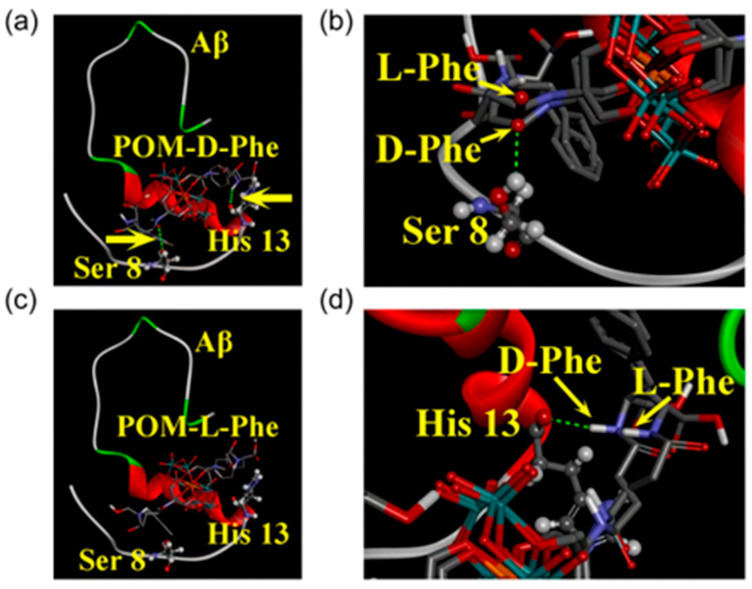
Energy-minimized average model of chiral POM derivatives-Aβ interactions. Chiral POM derivatives were visualized using a tube model, while the amino acid residues in Aβ were represented via a ball-and-stick model. (**a**) POM-D-phenylalanine-Aβ (POM-D-Phe-Aβ) complex: This complex gained additional stabilization through two hydrogen bonds, which were located at Ser 8 and His 13 and indicated by yellow arrows. (**b**) POM-L-phenylalanine-Aβ (POM-L-Phe-Aβ) complex: No hydrogen bond formation was observed in this complex. (**c**,**d**) Supplementary depictions of the two hydrogen bonds: These bonds were formed between POM-D-Phe and Aβ at Ser 8 (**c**) and His 13 (**d**). Atom colors: carbon (grey), oxygen (red), nitrogen (blue), manganese (orange), molybdenum (cyan) [35].

In 2019, Gao et al. designed and synthesized a series of chiral amino acid-modified polyoxometalate derivatives based on the chiral properties of Aβ peptides, including positively charged amino acids (D-Histidine, L-Histidine), negatively charged amino acids (D-Glutamic acid, L-Glutamic acid), and hydrophobic amino acids (D-Leucine, L-Leucine, D-Phenylalanine, L-Phenylalanine) (Figure 2d) [35]. They found that the synergy between hydrophobic interactions and hydrogen bonds is the key to achieving enantioselective in hibition: both POM-D-Phe: D-Phenylalanine-modified polyoxometalates and POM-L-Phe: L-Phenylalanine-modified polyoxometalates bind to the hydrophobic F19-F20 core of Aβ via hydrophobic interactions, disrupting inter-chain π–π stacking. However, the D-Phe moiety in POM-D-Phe forms two additional hydrogen bonds with the Ser8 hydroxyl group and the His13 imidazole group—interactions that are not attainable for POM-L-Phe (Figure 5). Consequently, the binding constant of POM-D-Phe toward Aβ is eightfold higher than that of POM-L-Phe. This discovery provides the first confirmation that chiral POMs can interfere with Aβ aggregation through enantioselective binding. Moreover, these derivatives exhibit favorable BBB permeability and low cytotoxicity even at elevated concentrations, offering a promising avenue for the development of chiral therapeutics.

In 2019, Zhao’s group designed an organoplatinum-substituted POM, (Me_4_N)_3_[PW_11_O_40_(SiC_3_H_6_NH_2_)_2_PtCl_2_] (abbreviated as Pt^II^-PW_11_) [51]. Pt^II^-PW_11_ binds to the positively charged HHQK region of Aβ_42_ via electrostatic attraction, and interacts with multiple residues of Aβ_42_ through van der Waals forces, hydrogen bonding, and desolvation effects. In addition, the Pt^2+^ center can coordinate with amino groups from residues such as His14 and Lys16 along the Aβ_42_ peptide chain. These multiple interactions collectively inhibit the nucleation of Aβ_42_ β-sheet structures, thereby suppressing fibril growth. The organoplatinum ligand of Pt^II^–PW_11_ adopts a planar conformation, enabling it to bind to Aβ_42_ aggregates and promote their dissociation. Compared with conventional POMs, the larger surface area of Ptᴵᴵ–PW_11_ further enhances its binding affinity toward Aβ_42_ through multi-point interactions. 

In 2022, our group designed and synthesized a novel polyoxometalate compound, (H_2_dap)_6_[CdCl_2_(B-α-AsW_9_O_34_)_2_]_8_·8H_2_O (dap = 1,2-diaminopropane, abbreviated as CdAW), which comprises rhombic tetranuclear cadmium clusters (Cd_4_Cl_2_O_14_) sandwiched between two trivacant Keggin-type arsenotungstate POMs to form a sandwich-like structure (Figure 2e) [52]. Owing to the synergistic effects arising from structural hybridization, CdAW modulates Aβ conformation through hydrogen bonding involving surface oxygen atoms of the POM framework and coordination between cadmium-cluster sites and histidine residues. In addition, it competitively inhibits Cu^2+^ binding to Aβ through the same coordination pathways, thereby reducing Cu^2+^-β complex formation and mitigating oxidative stress.

Protein post-translational modification (PTM) is a key mechanism for expanding proteome complexity in organisms [76]. PTMs involve covalent modifications at one or more specific amino acid residues after protein translation, resulting in substantial alterations to protein structure and function [77]. In the field of disease treatment, chemical PTM reagents target specific modification sites of proteins through covalent bonds, act precisely on specific modification sites or modification types, specifically regulate the functions of disease-related proteins, and reduce interference with normal physiological functions [78]. Based on this, Gao et al. proposed an innovative strategy of simulating natural PTM in 2022 and synthesized a TZ-modified POM derivative (POMD-TZ) (Figure 2f) [53]. Via the dual mechanism of POMD targeting the HHQK region and TZ covalently modifying the Lys16 site, site-selective covalent modification of Aβ was achieved, which markedly enhancing the intervention efficiency for Aβ aggregation. Incorporating the concept of chemical post-translational modification into POM design offers a direction for the simultaneous precise targeting and functional blocking of polyoxometalates in future designs. Notably, Gao’s group further developed POMD-TZ into POMD-Tar-TZ-FRET (Tar, Aβ-targeted peptide; FRET, Fluorescence Resonance Energy Transfer), which integrates Aβ-targeting capability with fluorescence-imaging functionality. This enables the specific recognition and visualization of highly toxic Aβ oligomers, further expanding the application scenarios of POMs in AD diagnosis and therapy.

**Figure 6:**
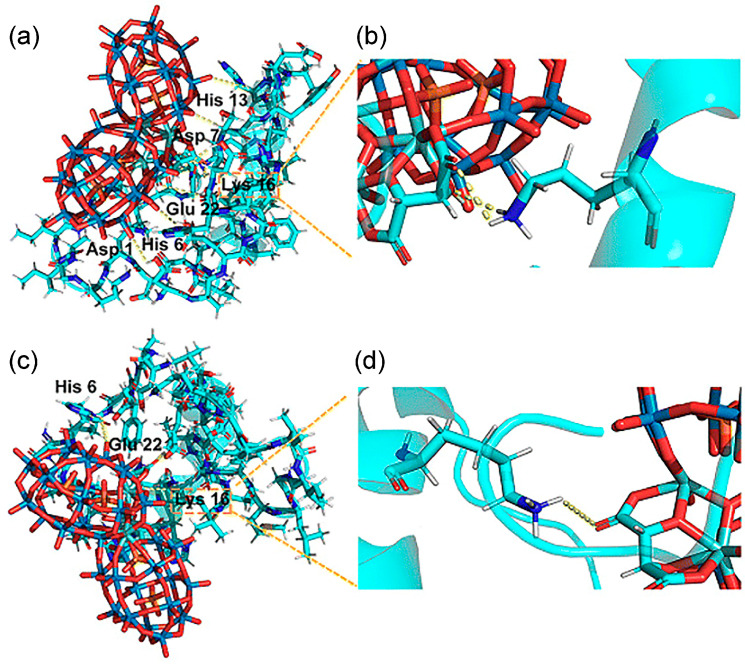
(**a**,**b**) The D-POM–Aβ complex (D-POM, D-enantiomeric polyoxometalate). Two hydrogen bonds were formed occurring at Lys 16, which are shown in partially enlarged views. (**c**,**d**) The L-POM–Aβ complex (L-POM, L-enantiomeric polyoxometalate). There is only one hydrogen bond between Lys 16 and L-POM [36].

In 2023 Hu et al. reported the synthesis of a pair of intrinsic enantiomeric POMs, [(CH_3_)_2_NH_2_]_15_{[α-P_2_W_15_O_55_(H_2_O)]Zr_3_(μ_3_-O)(H_2_O)(L-tartH)[α-P_2_W_16_O_59_]} and [(CH_3_)_2_NH_2_]_15_{[α-P_2_W_15_O_55_(H_2_O)]Zr_3_(μ_3_-O)(H_2_O)(D-tartH)[α-P_2_W_16_O_59_]} [36]. The nanoscale dimensions of these POMs are compatible with the α-helical segment of Aβ. They target the HHQK cationic cluster of Aβ_40_ via electrostatic interactions and simultaneously interfere with Aβ conformational transitions through hydrogen bonds, thereby blocking β-sheet formation and inhibiting Aβ aggregation. Due to chiral differences, D-POM forms two hydrogen bonds with Aβ Lys16, whereas L-POM forms only one hydrogen bond (Figure 6). This distinction yields a higher binding affinity of the D-enantiomer for Aβ_40_ relative to the L-enantiomer, resulting in more pronounced inhibition of Aβ fibrillation. This approach overcomes the limitation that earlier amino-acid-modified POMs required exogenous chiral units and offers a new direction for the structural design of POM-based materials. 

### 2.4. POM-Based Nanocomposites

Nanocomposite-based drug delivery systems hold considerable promise for targeted therapies by enabling the efficient delivery of therapeutics to specific sites while minimizing adverse effects on healthy tissues [79]. Incorporating POMs into nanocarriers through strategies such as self-assembly or ligand exchange not only reduces the intrinsic cytotoxicity of free POMs but also improves their stability under physiological conditions [80]. This integration strategy effectively shields the nonspecific interactions of POMs through surface modification and encapsulation mechanisms of nanocarriers, improving their dispersion and biocompatibility, thereby laying a solid foundation for the precise treatment of Alzheimer’s disease [81]. Moreover, nanocarrier-assisted controlled release technology and brain-targeted delivery strategies further optimize the balance between efficacy and safety, enhancing the effectiveness and acceptability of the therapeutic approach [82].

In 2013, the Qu group first constructed peptide-polyoxometalate-based hybrid nanospheres (POM@P) [54]. POM@P was formed by self-assembling K_8_[P_2_CoW_17_O_61_] with the Aβ-targeting short peptide Aβ_15–20_. This system can be regarded as a representative model in which POMs interact with the cationic clusters of Aβ through electrostatic attraction, while the Aβ_15–20_ peptide specifically binds to the hydrophobic core region of Aβ_1–40/42_; together, these interactions cooperatively inhibit Aβ aggregation. Studies have shown that the synergistic combination of these two interactions enables POM@P to significantly reduce Aβ aggregation by over 65% and to exhibit favorable safety by increasing cell viability to around 82%. This material exhibits favorable stability in mouse cerebrospinal fluid, showing a POM release rate of only 24.7% over 48 h, providing an important reference for the construction of long-acting delivery systems.

In 2014, the Gao group developed the multifunctional nanocomposite AuNPs@POMD-pep (AuNPs: gold nanoparticles; POMD: polyoxometalate with a Wells–Dawson structure; pep: peptide), extending its anti-Aβ mechanism from single aggregation blocking to a synergistic pathway combining aggregation suppression and depolymerization of preformed fibrils [55]. The LPFFD peptide binds Aβ through hydrophobic interactions and promotes fibril depolymerization, while POMD targets the adjacent HHQK cationic cluster (His13–Lys16) via electrostatic attraction and hydrogen bonding. Together, these two components broaden the effective binding region to His13–Phe20, resulting in synergistic inhibition of Aβ aggregation. Compared with POM@P, AuNPs@POMD-pep integrates dual inhibitory mechanisms, with AuNPs enhancing POM dispersion and improving blood–brain barrier penetration. This system reduces Aβ aggregation by approximately 47% while maintaining high cell viability (>90%).

In 2019, the Liu group developed Aβ-targeting peptide-modified molybdenum-based polyoxometalate nanoparticles (Peptide@Mo-POMs) to address Zn^2+^-induced Aβ40 aggregation through a dual synergistic mechanism combining competitive occupation of Zn^2+^-binding sites on Aβ and high-affinity chelation of free Zn^2+^ ions by the polyanionic Mo-POM scaffold [37]. This design retains the electrostatic and hydrophobic recognition features of earlier peptide–POM systems while introducing metal ion chelation as an additional inhibitory pathway. Peptide@Mo-POMs exhibit good biocompatibility and targeting specificity, significantly enhancing pheochromocytoma cell line (PC12) cell viability in a Zn^2+^-induced Aβ40 injury model and reducing apoptosis and intracellular ROS levels. Transwell assays further confirmed their effective blood–brain barrier penetration, supporting their potential for in vivo application.

In 2022, Govindaraju et al. developed a cyclic dipeptide-polyoxometalate (CP-POM) nanocomposite by integrating a Keggin-type POM with a CDP-CP through hydrogen-bond-driven coacervation [56]. In this system, the cyclic dipeptide copolymer self-assembles into stable nanostructures through noncovalent interactions, contributing to enhanced biocompatibility and structural integrity of the material. Compared with conventional POM-based nanocomposites, CP-POM modulates Aβ aggregation via weak noncovalent interactions and, owing to the redox activity of POM, exhibits pronounced ROS scavenging capability, thereby alleviating Aβ-induced oxidative stress and cytotoxicity. This synergistic, multi-mechanistic action provides a new strategy and expands the functional potential of POM-based nanomaterials for Alzheimer’s disease intervention.

**Figure 7:**
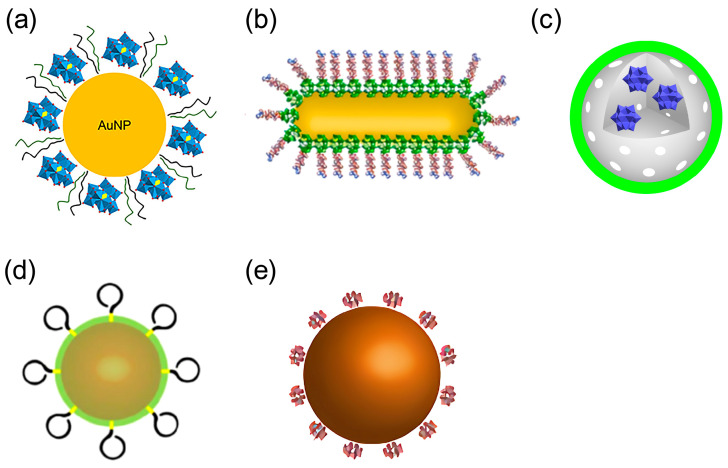
Structures of POM-based nanocomposites. (**a**) AuNPs@POM@PEG (AuNPs, gold nanoparticles; POMs, polyoxometalates; PEG, polyethylene glycol) (**b**) peptide conjugated Au nanorods (AuP) (**c**) rPOMs@MSNs@copolymers (rPOMDs, reduced polyoxometalates with Wells–Dawson structure; MSNs: mesoporous silica nanoparticles, copolymer: poly(N-isopropylacrylamide-co-acrylamide)) (**d**) AuNPs@POMD-8pep(AuNPs, gold nanoparticles; POMD, Wells–Dawson polyoxometalate, 8pep: N-Ac-Cys-hepta-peptide) (**e**) cerium dioxide/polyoxometalate (CeONP@POMs).

In 2023, Perxés Perich and colleagues developed a tri-component nanosystem, AuNPs@POM@PEG, comprising gold nanoparticles (AuNPs), polyoxometalates (POMs), and polyethylene glycol (PEG) for Aβ aggregation inhibition and brain-targeted delivery (Figure 7a) [38]. In this nanosystem, AuNPs act as carriers that promote uniform POM dispersion; POMs disrupt the conformational transitions of Aβ monomers through electrostatic interactions and steric hindrance; and PEG reduces nonspecific protein adsorption and prolongs systemic circulation via steric stabilization. Collectively, these mechanisms result in approximately 75% inhibition of Aβ aggregation in vitro and demonstrate good biocompatibility at concentrations below 2.5 nM, alongside effective penetration in an in vitro BBB model. Compared with earlier peptide-POM nanocomposites, AuNPs@POM@PEG shows improved dispersion, aggregation inhibition, circulation time, and BBB targeting. However, in vitro BBB models may not fully reflect in vivo transport, and the long-term toxicity of gold carriers remains unclear. Despite these limitations, this work illustrates the trend toward multifunctional POM-based nanotherapeutics for AD and underscores the need for rigorous mechanistic and comparative evaluation.

Overall, POM-based nanocomposites have progressed from simple peptide-functionalized systems to multifunctional constructs that combine electrostatic and hydrophobic interactions, metal ion chelation, redox activity, and carrier-mediated delivery. This evolution has enhanced their stability under physiological conditions, reduced cytotoxicity, and broadened mechanistic versatility. Nonetheless, systematic studies addressing long-term toxicity, dose-dependence, in vivo biodistribution, and effective blood–brain barrier penetration remain limited. To advance POM-based nanotherapeutics, future research should focus on standardized quantitative comparisons, comprehensive safety evaluations, and rigorous assessment of pharmacokinetics and BBB transport, enabling mechanism-guided design and improving translational potential. Collectively, these developments underscore both the promise and the challenges of applying multifunctional POM-based nanomaterials for Alzheimer’s disease therapy. 

In conclusion, POMs exhibit a pronounced structure-dependent profile in both their toxicity and selectivity toward Aβ. Key structural parameters influencing these properties include surface charge density, metal composition and oxidation state, molecular size and geometry, as well as the nature of ligands or functional groups [31]. For purely inorganic POMs, higher negative charge density and larger cluster structures enhance electrostatic interactions with positively charged residues in Aβ (e.g., the His13–Lys16 region), thereby inhibiting fibril formation, but simultaneously increase non-specific interactions with other biomolecules, leading to higher cytotoxicity. In contrast, modifications such as transition-metal substitution or organic ligand functionalization can fine-tune the charge distribution and coordination environment, introducing specific coordination and hydrophobic interactions that improve selective binding to Aβ while mitigating off-target interactions and cytotoxicity. Moreover, assembling POMs into hybrid materials or nanocomposites can further reduce toxicity by stabilizing cluster structures and minimizing direct exposure of highly charged inorganic surfaces to the biological milieu, thus enhancing biocompatibility and therapeutic potential.

## 3. Application of POMs in Phototherapy

Phototherapies have emerged as important approaches to overcome the limitations of traditional treatments in the research on non-invasive AD therapy, owing to their core advantages of precise spatiotemporal control and minimal damage to normal tissues [83,84]. POMs exhibit tunable redox properties and favorable optical response characteristics, making them well suited for integration into multiple phototherapeutic mechanisms [85,86], and thereby serving as valuable components for constructing AD-targeted phototherapeutic systems [58].

### 3.1. Application of POMs in Photodynamic Therapy (PDT)

The therapeutic basis of photodynamic therapy (PDT) lies in the selective elimination of pathological substrates through light-triggered ROS generation, a mechanism that holds distinct value in AD-related pathological intervention [87]. The key to this therapy lies in the efficient ROS generation by photosensitizers under specific light excitation [88,89]. Certain POMs exhibit strong ultraviolet absorption, display photocatalytic activity, and generate ROS through photocatalytic water splitting [90,91].

**Figure 8:**
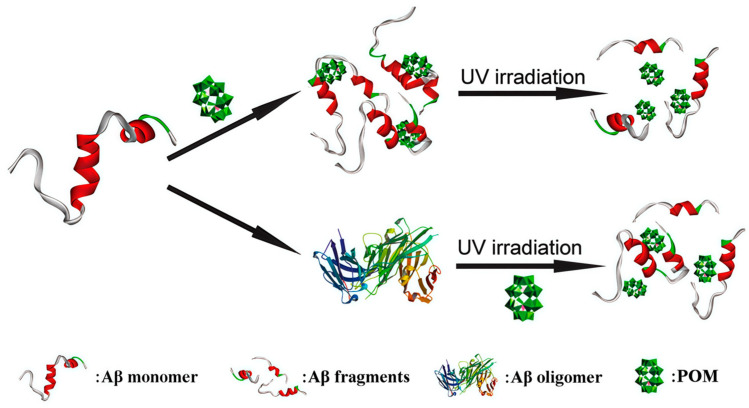
Schematicrepresentation of POMs used to effectively inhibit the aggregation of Aβ peptides by degrading their monomers and oligomers upon photo-irradiation [46].

In 2013, Li et al. first demonstrated that a Wells-Dawson-type polyoxometalate, K_8_[P_2_CoW_17_O_61_], not only inhibits Aβ aggregation but also promotes the degradation of β-sheet-rich Aβ assemblies under UV irradiation via photocatalysis (Figure 8) [46]. The UV-activated POM generates ROS, which disrupts hydrogen bonding in Aβ and destabilizes β-sheet structures, thereby facilitating the breakdown of oligomers and fibrils. Despite confirming the feasibility of POM-mediated photocatalytic degradation of pathological Aβ, the limited tissue penetration of UV light restricts in vivo applicability, highlighting the need to develop near-infrared (NIR)-responsive POMs for deeper tissue penetration and enhanced therapeutic potential. 

### 3.2. Application of POMs in Photothermal Therapy (PTT)

Photothermal therapy (PTT) is a minimally invasive or non-inasive therapeutic modality that achieves targeted effects on the affected area via precise light control, minimizing systemic side effects and damage to surrounding healthy tissues [92]. Currently, PTT-based AD therapy focuses on disrupting preformed Aβ aggregates or inhibiting their abnormal aggregation via the photothermal effect of photothermal transducers (PTAs) [93,94,95]. Owing to their tunable structures and dual optical-biological activities, POMs exhibit unique advantages in photothermal AD therapy. In recent years, studies have further expanded the functional scope of POM-based phototherapeutic systems. Composite systems constructed by combining POMs with nanocarriers can not only leverage the deep tissue penetration of NIR light to address the poor penetration of traditional ultraviolet light but also enhance BBB delivery efficiency via carrier modification [96].

**Figure 9:**
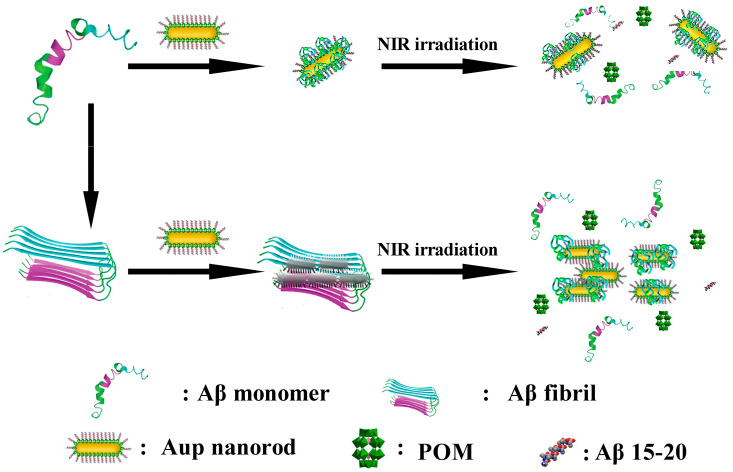
Schematic representation of the peptide conjugated AuP nanorods used for Alzheimer’s disease treatment [57].

In 2017, Li et al. developed a nanoprobe based on peptide conjugated Au nanorods (AuP) [57]. AuP was obtained by co-assembling POM and the Aβ_15–20_ peptide onto the surface of gold nanorods with strong NIR absorption features via a self-assembly process (Figure 7b). Benefiting from the localized surface plasmon resonance of gold nanorods, AuP exhibits strong absorption in the near-infrared region at 808 nm and efficiently converts incident light into localized heat under irradiation. This photothermal effect perturbs pre-formed Aβ fibrils, thereby synergistically enhancing the inhibitory activity of POMs and Aβ_15–20_ peptides against Aβ aggregation, highlighting the auxiliary role of photothermal stimulation in regulating the dynamic assembly of Aβ (Figure 9). Additionally, binding of AuP to Aβ fibrils leads to the aggregation of nanorods, accompanied by a blue shift of the longitudinal LSPR peak and a red shift of the transverse peak. The degree of spectral change is positively correlated with the amount of Aβ aggregates, enabling the quantitative detection of Aβ aggregates. AuP exhibits BBB penetration, with a brain gold accumulation rate of 2.097 ± 0.337%, providing crucial delivery feasibility to support its in vivo diagnostic and therapeutic application in Alzheimer’s disease. Collectively, this work establishes a theranostic nanoplatform that integrates the detection, inhibition, and photothermal dissociation of Aβ aggregates, highlighting the potential of POM-based hybrid systems for Alzheimer’s disease diagnosis and therapy. 

In 2018, Ma et al. reported a redox-activated NIR-responsive nanoplatform, rPOMDs@MSNs@copolymers (rPOMDs: reduced polyoxometalates with Wells–Dawson structure; MSNs: mesoporous silica nanoparticles; copolymer: poly(N-isopropylacrylamide-co-acrylamide)) (Figure 7c), which integrates Aβ aggregation inhibition, fibril disaggregation, and ROS scavenging within a single photothermal system [58]. Under 808 nm laser irradiation, rPOMDs generate localized hyperthermia via the photothermal effect, which not only directly disaggregates Aβ fibrils but also melts the copolymer film to release rPOMDs. After release, the rPOMDs scavenge Aβ-induced ROS by virtue of their redox activity, reducing the ROS level by approximately 52.8%. Meanwhile, they directly inhibit the aggregation and fibrillogenesis of Aβ monomers, resulting in an inhibition rate of 74.7% for Aβ aggregation by rPOMDs@MSNs@copolymer under NIR light activation. In addition, this system protects cells from Aβ-induced cytotoxicity, restoring cell viability to over 90%. Furthermore, even after oxidation to POMs, they can still continuously inhibit Aβ aggregation.

Reviewing current studies, POM-based photodynamic therapy and photothermal therapy offer potentially complementary strategies for non-invasive therapeutic research in Alzheimer’s disease. PDT relies on ultraviolet-light-activated POMs to generate ROS, which can effectively degrade Aβ aggregates, but its application is limited by the poor tissue penetration of ultraviolet light and the wavelength-dependent efficiency of photocatalysis. In contrast, PTT employs NIR-responsive POM composites, enabling deeper tissue penetration and efficient photothermal conversion—AuP can achieve effective dissociation of Aβ fibrils under 1 W/cm^2^ irradiation, while rPOMDs@MSNs@copolymer exhibits pronounced fibril dissociation after 8 min of irradiation at 1.8 W/cm^2^. Multiple studies indicate that specific POM materials possess potential in ROS generation, photothermal conversion, and modulation of Aβ aggregation; however, these findings are largely based on in vitro and animal models and require further validation. Future research should focus on optimizing NIR-responsive POM designs, improving therapeutic safety, and establishing standardized evaluation metrics to facilitate clinically translatable development.

## 4. Application of POMs in Artificial Proteases

In AD therapy-related studies, although POMs can interfere with pathological progression by inhibiting Aβ aggregation or via phototherapy, these strategies only alter the ratio of aggregated to non-aggregated Aβ forms without reducing the total Aβ load [97]. In contrast, artificial proteases based on metal active centers can fundamentally reduce Aβ levels by hydrolyzing Aβ peptide bonds, offering a more direct solution for AD therapy. Introducing metal ions with high coordination numbers and pronounced Lewis acidity (e.g., Hf^4+^, Zr^4+^, Ce^4+^) into lacunary POM structures enables the formation of metal-substituted POMs (MS-POMs) with protease-like activity [98,99]. MS-POMs mainly bind through electrostatic attraction between their negatively charged surfaces and positively charged protein regions, whereas the Lewis-acidic metal centers facilitate hydrolysis of peptide bonds [98,99,100]. Among reported MS-POMs, Hf^4+^-, Zr^4+^-, and Ce^4+^-based systems generally show higher peptide-bond hydrolysis activity, which correlates with the Lewis acidity of these +4 oxidation state ions and their relatively high coordination numbers (e.g., Ce^4+^ up to 12; Hf^4+^ and Zr^4+^ commonly around 8) [101,102]. Notably, unlike Hf^4+^ and Zr^4+^, Ce^4+^ may exhibit redox activity during the hydrolysis reaction [102]. However, studies applying MS-POMs to Aβ hydrolysis remain limited, and existing work has largely concentrated on nanocomposite-based designs. Improving Aβ-binding selectivity together with system stability and biocompatibility through nanocomposite construction has become an important direction in advancing MS-POM-based artificial proteases for intervention in Aβ pathology in AD [59].

In 2016, building on AuNPs@POMD-pep, the Qu group further optimized the design and constructed a nanozyme, AuNPs@POMD-8pep (AuNPs: gold nanoparticles; POMD: Wells–Dawson polyoxometalate; 8pep: N-Ac-Cys-hepta-peptide) (Figure 7d), with protease-like activity, superoxide dismutase (SOD)-like activity, and Cu^2+^-chelating ability via the introduction of a histidine-rich octapeptide (8pep) [59]. This material inherits the BBB penetration advantage of gold nanoparticles while achieving a key functional breakthrough.

**Figure 10:**
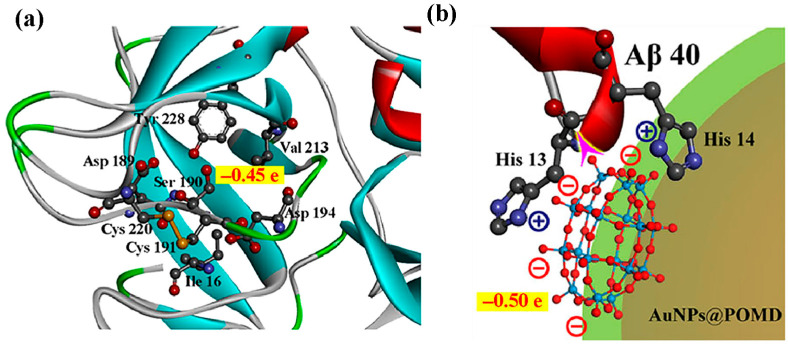
(**a**) Mulliken charge distributions for the Ser190 hydroxyl group in native protease, and (**b**) the oxygen groups of POM in the nanozyme. The purple arrow in (**b**) illustrates the electronegative oxygen groups of POM attacking the electropositive carbon atom in the peptide bond [59].

**Figure 11:**
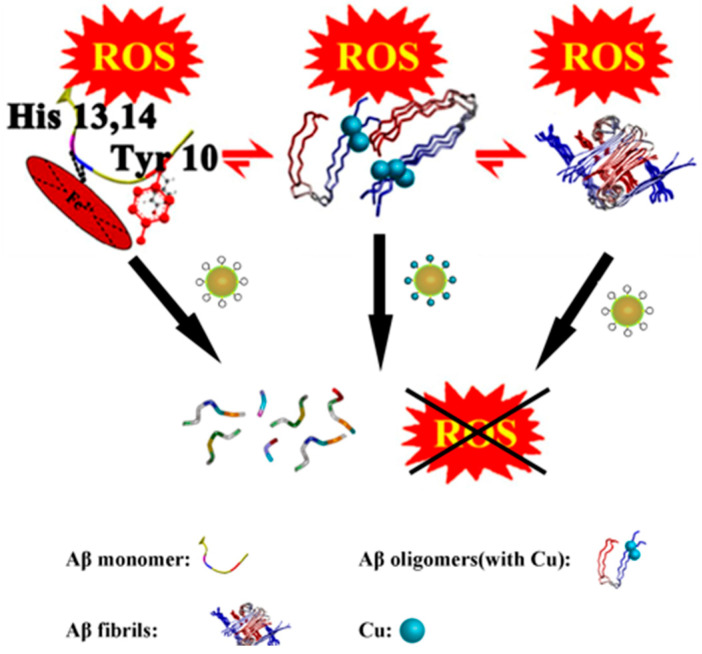
AuNPs@POMD-8pep acted as a multifunctional nanozyme to modulate multiple facets of Alzheimer’s disease [59].

The negatively charged oxygen groups of POMD can mimic the negatively charged hydroxyl groups in natural serine proteases, acting as nucleophiles to attack the carbonyl carbon atoms of Aβ peptide bonds, thereby directly hydrolyzing Aβ monomers and aggregates (Figure 10). This addresses the limitation of AuNPs@POMD-pep, which inhibits Aβ aggregation but does not remove pre-existing aggregates. In addition, nitrogen atoms in histidine residues of 8pep coordinate with Cu^2+^ to form a SOD-like catalytic site, contributing to removal of Aβ-associated ROS through electron transfer. Meanwhile, this process also binds free Cu^2+^, mitigating Cu^2+^-induced aggregation of Aβ and providing combined modulation of metal imbalance and oxidative stress (Figure 11). Subsequently, the Gao group further introduced the Aβ-targeting LPFFD hexapeptide (6pep) to construct AuNPs@POMD-8pep-6pep, which further enhances the nanozyme’s Aβ targeting ability.

In 2016, the Qu group reported a bifunctional artificial nanozyme with protein hydrolysis and SOD activity—cerium dioxide/polyoxometalate (CeONP@POMs) nanoparticles (Figure 7e) [60]. In this system, POMs bind electrostatically to the cationic clusters (HHQK region) of Aβ monomers and protofibrils via their negatively charged surfaces.

**Figure 12:**
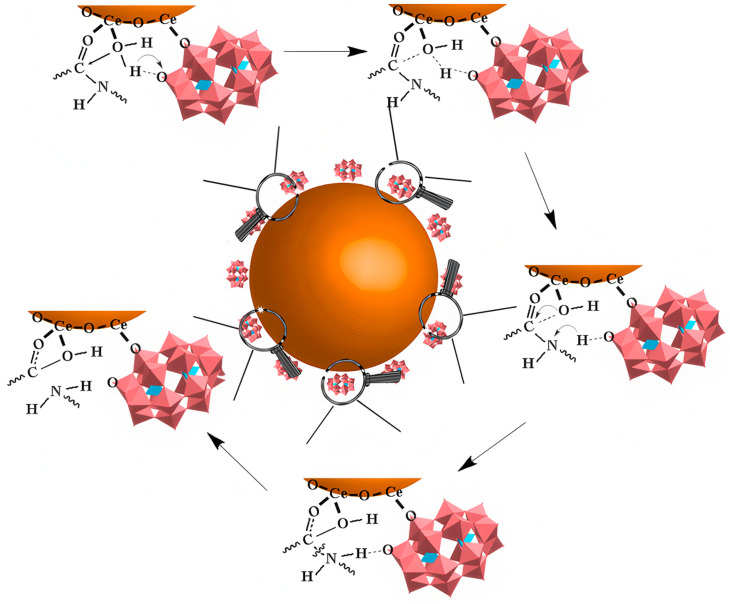
The catalytic mechanism of CeONP@POMs. The brown sphere represents the CeONP core, the pink structures indicate the POMs coating on the surface. and the arrow denotes the hydrolytic degradation process [60].

During this process, driven by the interaction between POMs and Aβ, Ce^4+^ in CeONPs approaches the peptide bonds of Aβ monomers and coordinates with the carbonyl oxygen atoms of these bonds. This coordination polarizes the carbonyl carbon, making it more susceptible to nucleophilic attack by water molecules and thereby promoting peptide-bond hydrolysis (Figure 12).

**Figure 13:**
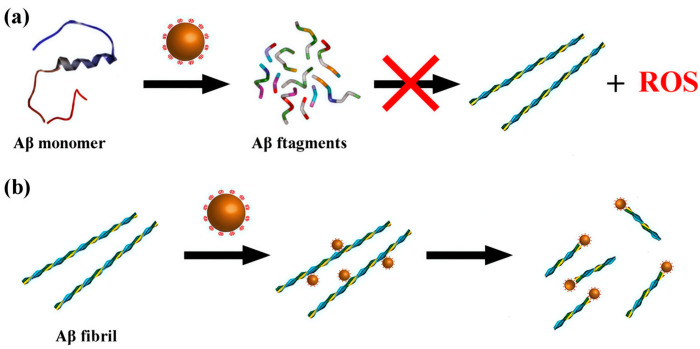
Scheme of CeONP@POMD as hydrolytic enzyme to degrade Abβ_40_ monomers (**a**) and Aβ_40_ fibrils (**b**). Brown particles represent the CeONP@POMD core (see Figure 12 caption for details), while the other structures in different colors represent Aβ monomers, Aβ fibrils, and their degradation fragments. Arrows indicate the hydrolytic degradation process [60].

Simultaneously, the electrostatic interaction between POMs and Aβ fibrils disrupts the intermolecular hydrogen bonds and hydrophobic interactions within the fibrils, which impairs fibril stability and induces the depolymerization of Aβ fibrils into oligomers or monomers. The exposed peptide bonds are then hydrolyzed (Figure 13). Regarding the antioxidant mechanism, Ce^3+^ and Ce^4+^ on the surface of CeONPs scavenge ROS via rapid electron transfer, enabling CeONP@POMs to exhibit SOD activity.

Despite both AuNPs@POMD-8pep and CeONP@POMs degrading Aβ and scavenging ROS, their mechanisms differ: AuNPs@POMD-8pep targets the Aβ HHQK region and regulates metals via His-Cu^2+^ coordination, whereas CeONP@POMs relies mainly on Ce^3+^/Ce^4+^ electron transfer. AuNPs@POMD-8pep shows markedly superior proteolytic performance (specific activity 8.80 × 10^5^ U/mg vs. commercial trypsin’s 5.14 × 10^5^ U/mg; better kinetics), enabling faster Aβ clearance at lower doses. CeONP@POMs has lower activity (64.71 U/mg) but superior physiological stability. Both offer comparable SOD-like activity and ROS clearance, yet AuNPs@POMD-8pep gains extra multi-target benefits from Cu chelation against AD metal dysregulation. Both nanoplatforms exhibited good biocompatibility and blood–brain barrier (BBB) penetration in animal models. Overall, AuNPs@POMD-8pep excels in efficiency and multifunctionality for rapid clearance scenarios, while CeONP@POMs, with simpler design, better stability, and neurorepair potential, suits milder physiological or regenerative needs. Head-to-head animal studies will clarify clinical potential; these works demonstrate artificial nanozymes’ major advantage over natural enzymes and provide key directions for multi-target AD nanotherapies, while highlighting remaining challenges such as long-term stability, potential toxicity, BBB penetration, and translational applicability. 

**Figure 14:**
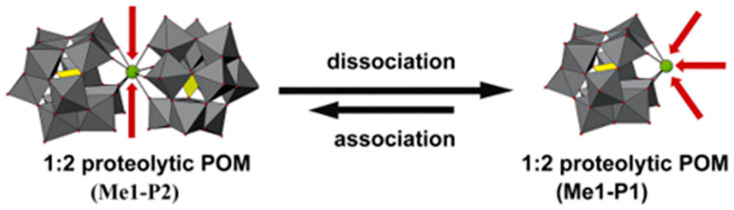
1:2 proteolytic POM (Me1-P2; Me = metal, P = POM) hydrolysis may dissociate into 1:1 proteolytic POM (Me1-P1). Me1-P2 consists of one hydrolytically active metal (green sphere, Me1) and two Keggin-type POMs (addenda atoms are depicted as gray polyhedra, oxygen atoms as small red spheres, and the incorporated heteroatoms as yellow polyhedra), and can dissociate into Me1-K1 under specific conditions, potentially reassociating. In Me1-P2, the active metal is shielded, resulting in low hydrolytic activity, whereas after dissociation into Me1-P1, the metal is exposed and hydrolytic activity is enhanced [105].

Studies have further shown that the Aβ hydrolytic activity in this system follows the order: Wells-Dawson-type POM > Keggin-type POM > Anderson-type POM.The core reason is that Wells-Dawson type POM possesses a higher surface negative charge density, a molecular size more matching the Aβ binding sites, and a superior regulatory effect on the Lewis acidity of Ce^4+^. Furthermore, other studies have further revealed that the configuration of POMs and the activity characteristics of Lewis acidic metal ions are also key factors regulating hydrolysis efficiency [103,104,105,106,107]. POM composites containing one or more Lewis acidic metals or composed of one or more POMs (e.g., M_IV2_-POM_2_, where M = Zr, Hf, Ce) tend to dissociate in solution to form their monomeric composites with hydrolysis activity (M_IV1_-POM_1_) (Figure 14) [108]. In the 1:1 monomer, Lewis acidic metals (e.g., Zr^4+^ and Ce^4+^) are more fully exposed and have more free coordination sites, which can directly coordinate with the carbonyl oxygen atoms of amide bonds [104,105,107,108]. Consequently, stronger Lewis acidity and greater availability of coordination sites generally correlate with higher hydrolytic activity. Establishing the relationship between POM structure and hydrolytic activity offers guidance for structural refinement of POM-based artificial proteases with enhanced catalytic performance.

## 5. Progress and Application of POMs to Other Target Proteins in Alzheimer’s Disease Treatment

The pathological process of AD is not driven by a single factor but involves a complex network of multiple pathways, including neuroinflammation activation and cholinergic system dysfunction [109]. In addition to Aβ, the inflammation-associated protein S100A9 and cholinesterases (AChE and BChE) also contribute to disease progression and are considered potential targets for POM-mediated intervention.

### 5.1. Targeting S100A9

**Figure 15:**
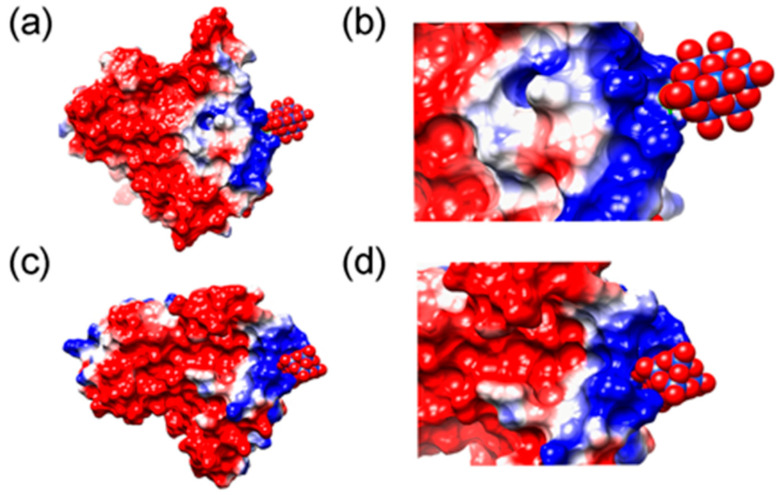
MD simulation of interactions of S100 calcium-binding protein A9 with POMs. (**a**,**c**) Binding of Nb_10_ and TiNb_9_, respectively, to a subunit of S100A9 dimer. The electrostatic potentials on the protein surface are colored by a red−white−blue gradient with the values spanning from −5.0 to 5.0 kT/e. Nb_10_ and TiNb_9_ are shown as van der Waals spheres, where oxygen is presented in red, covering Nb atoms shown in blue and Ti in pink. (**b**,**d**) Magnified views of the complex formation of Nb_10_ and TiNb_9_, respectively, on the S100A9 surface based on ionic interactions [61].

The amyloid–neuroinflammatory cascade represents an important pathological mechanism in Alzheimer’s disease [110]. S100A9, a pro-inflammatory calcium-binding protein, has been implicated in this process [111]. It is expressed by neurons and microglia and, through binding to receptors including the receptor for advanced glycosylation end-products (RAGE) and Toll-like receptor 4 (TLR4), induces the expression of pro-inflammatory cytokines and activates inflammatory signaling pathways, thus exacerbating the neuroinflammatory response [112]. Compared with other pro-inflammatory mediators, S100A9 is characterized by high amyloidogenicity that initiates and promotes the self-assembly of Aβ amyloid and exacerbates amyloid-associated cytotoxicity [113,114]. Therefore, inhibiting the amyloid aggregation and pro-inflammatory functions of S100A9 has emerged as an important strategy for intervening in the pathological process of Alzheimer’s disease. In 2021, Chaudhary et al. demonstrated that two niobium-based POM nanomaterials (Nb_10_ and TiNb_9_) bind to the positively charged, lysine (Lys)-rich regions on the surface of S100A9 via electrostatic interactions [61]. This binding induces only local conformational changes at the binding site without causing large-scale perturbations to the secondary or tertiary structure of S100A9, while it can effectively inhibit its amyloid aggregation (Figure 15). The binding region (Lys50-Lys54) exhibits high amyloid propensity and is recognized as a key sequence involved in the amyloid self-assembly of S100A9. Thus, targeting this region is considered a potential approach to modulate the S100A9-driven amyloid-neuroinflammatory cascade.

### 5.2. Targeted Cholinesterase (CHE)

In the cholinergic hypothesis, patients with Alzheimer’s disease exhibit a significant reduction in cerebral acetylcholine levels due to factors including the degeneration of cholinergic neurons and decreased acetylcholine synthesis, which constitutes one of the key mechanisms underlying cognitive dysfunction [115,116]. In addition, hydrolysis of acetylcholine by acetylcholinesterase (AChE) and butyrylcholinesterase (BChE) further contributes to reduced acetylcholine levels [117,118]. Therefore, cholinesterase inhibitors can attenuate this process, thereby increasing acetylcholine levels and improving cognitive function in patients [119,120,121,122].

In 2012, Iqbal et al. first reported that POMs bind to AChE and BChE through electrostatic interactions. This binding induces conformational changes in the active sites of these enzymes, thereby inhibiting their catalytic hydrolysis activity via a non-competitive inhibition mechanism [41]. This study demonstrated for the first time that POMs can act as bifunctional inhibitors of AChE and BChE, providing new ideas for AD therapy. In 2017, Mirjana B et al. reported that two Keggin-type heteropolytungstates, K_7_[Ti_2_PW_10_O_40_]·6H_2_O(K-Ti_2_PW_10_) and K_6_H[SiV_3_W_9_O_40_]·3H_2_O (K-SiV_3_W_9_), exhibit distinct inhibitory activities against AChE. This finding provides important references for the development of POMs as cholinesterase inhibitors [62].

**Figure 16:**
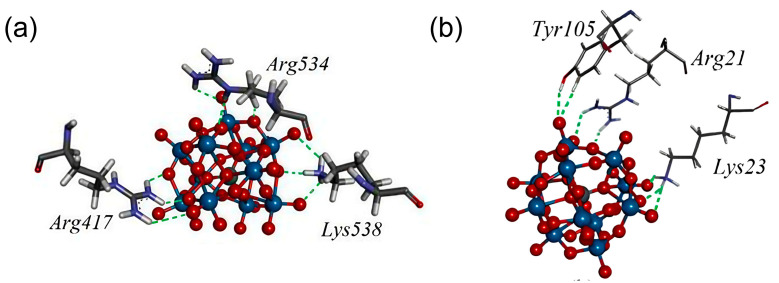
The amino acid environment of [SiW_11_O_39_]^7−^ in the most stable binding sites (BS1) at AChE, with closed-form (**a**) and open form (**b**) of the enzyme. Amino acid residues Arg21, Lys23, and Tyr105 form new allosteric site termed β-allosteric site (β-AS) [63].

In a recent study, Bondžić et al. examined the mechanism underlying POM-induced inhibition of AChE. This study was the first to reveal that AChE possesses a β-amyloid–related allosteric site (β-AS), which serves as the binding region for large, highly negatively charged POMs [63]. This site is located in the β-strand region (e.g., the Gln16–Pro25 fragment) on the AChE surface (Figure 16). POMs can specifically bind to the positively charged amino acid residues of β-AS via electrostatic interactions mediated by their negatively charged clusters. This process not only inhibits AChE activity but also prevents the conversion of the C-terminal oligomerization domain (AChE586–599) of AChE from an α-helical to a non-native β-sheet conformation by stabilizing the enzyme conformation, thus inhibiting AChE-mediated β-amyloid aggregation. This study highlights the potential of POMs as acetylcholinesterase inhibitors, providing a mechanistic information and valuable practical guidance for the future design and synthesis of POMs with enhanced cholinesterase inhibitory activity. 

## 6. Conclusions and Future Directions

The pathogenesis of AD involves intricate and interconnected pathological processes, with abnormal Aβ deposition recognized as a core hallmark [123]. Research on POMs in AD therapy has primarily focused on Aβ targeting, which employs multiple mechanisms including electrostatic interactions, van der Waals forces, and hydrogen bonding to inhibit Aβ aggregation and mediate Aβ fibril hydrolysis. To enhance Aβ recognition and inhibition, structural modifications of POMs have been explored, including transition metal substitution (e.g., Ni^2+^, Co^2+^) which alters surface charge density and introduces coordination with histidine residues to strengthen binding affinity; peptide functionalization which endows precise targeting via hydrogen bonding and electrostatic interactions with key Aβ domains; chiral ligand modification which confers enantioselective binding to the Aβ; and conjugation with nanoparticles (e.g., AuNPs) which enhances the hydrolysis efficiency of Aβ fibrils via synergistic Lewis acid catalysis. Beyond Aβ targeting, POMs have also been examined in phototherapeutic applications. As photocatalysts, they generate ROS under light irradiation to disrupt Aβ fibrils; as near-infrared photothermal agents, they disaggregate preformed Aβ aggregates via localized hyperthermia. In addition, POMs have been studied in relation to other molecular targets relevant to AD pathology. POMs interact with S100A9 and inhibit its amyloid assembly, which may modulate related neuroinflammatory responses. POMs can also bind to the β-AS of AChE, reduce enzyme activity through electrostatic interactions, attenuate acetylcholine hydrolysis, and increase acetylcholine availability. Existing evidence indicates that POMs may intervene in multiple pathological processes of AD through inhibiting Aβ aggregation, modulating inflammation-associated proteins, and influencing neurotransmitter metabolism. However, despite encouraging experimental progress, clinical translation of POM-based agents remains challenging [42]. The toxicity and biocompatibility of POMs need to be further optimized to ensure their safety and stability in vivo [124]. Meanwhile, improving the binding specificity of POMs to Aβ and other targets is also an important direction of current research, which requires more in-depth exploration of the specific mechanism of action between POMs and various targets. Additionally, in vivo pharmacokinetic assessment and translational evaluation of POM-based candidates require further efforts to support advancement from preclinical to clinical research [125].

Recently, researchers have predicted the binding modes and affinities of POMs to the 10 key proteins most closely associated with AD (including BACE1, PSEN1, PSEN2, APP, APOE, MAPT, SNCA, AChE, BChE, and BDNF) via molecular docking techniques [2]. Molecular docking studies confirm that POMs can bind to multiple key targets in AD pathology, including Aβ, cholinesterases, secretases, and APOE4, with their mechanisms of action involving the inhibition of Aβ aggregation, reduction in Aβ production, inhibition of cholinesterase activity, and enhancement of neuroprotection via the stabilization of brain-derived neurotrophic factor (BDNF). Theoretically, multi-mechanistic intervention in the pathological progression of AD can be achieved. With an improved understanding of the interaction mechanisms between POMs and AD-related targets, it is foreseeable that future research will focus on optimizing POM structures to enhance target selectivity and developing POMs with multifunctional therapeutic effects. Additionally, further exploration of the combined application of POMs with other AD therapeutic strategies (e.g., phototherapy, gene therapy) will be another important direction for future research.

In conclusion, POMs hold broad application prospects in Alzheimer’s disease therapy. Future studies should continue to elucidate the interaction mechanisms between POMs and pathogenic proteins, reinforce preclinical translation, including pharmacological validation and toxicity assessment, and explore emerging AI-assisted computational design strategies. Such AI-assisted approaches have the potential to go beyond conventional molecular docking, enabling more efficient optimization of POM structures and improving target selectivity toward AD-related proteins [126]. Additionally, the potential synergistic effects of POMs with other therapeutic modalities should be investigated to further enhance overall therapeutic performance. Trough continuous innovation and optimization, POMs are anticipated to emerge as a novel intervention strategy in the field of AD therapy with both innovative and clinical value, paving new avenues for improving cognitive function in patients.

## Figures and Tables

**Table 1:** POMs in the treatment of Alzheimer’s disease.

Category	Protein Aggregates	Name	Molecular Mechanism of Action	Quantitative Data	Multi-Target Effects	Ref.
1	Aβ	K_8_[P_2_CoW_17_O_61_]	Binds to Aβ through electrostatic interactions, generates ROS upon light excitation, and disrupts Aβ hydrogen bonds and secondary structure.	DCF fluorescence intensity increased by 163% (8 μM).		[32,46]
2	Aβ	Na_9_H[SiW_9_O_34_], K_7_[PTi_2_W_10_O_40_], K_8_[β-SiW_11_O_39_]	POMs binds to the His13-Lys16 cationic region of Aβ through electrostatic interactions, thereby inhibiting further Aβ aggregation.	Na_9_H[SiW_9_O_34_]: Aβ inhibition IC_50_ = 19.85 μM (in vitro, ThT); K_7_[PTi_2_W_10_O_40_]: Aβ inhibition IC_50_ = 39.04 μM (in vitro, ThT); K_8_[β-SiW_11_O_39_]: Aβ inhibition IC_50_ = 39.02 μM (in vitro, ThT).		[32]
3	Aβ	(NH_4_)_42_{Mo_132_O_372_(OAc)_30_} *	By binding through negative charges to the positively charged His13-Lys16 region of Aβ, and simultaneously chelating Zn^2+^/Cu^2+^ to reduce their concentration, it achieves dual inhibition of Aβ aggregation.	ROS reduction: 34% (10 μg/mL); Cell viability: >50% (10 μg/mL).	Inhibition of Aβ aggregation + metal ion chelation	[47]
4	Aβ	[PO_40_Mo_12_]^3−^	Inhibition of Aβ fibrils formation via multi-weak interactions (H-bonds, van der Waals).	Binding energy: −1.1 to −1.6 eV, ≈ −5 eV; Energy difference: ≥1–3 eV; Orbital gap: ≈ 2.1 eV (P–Iso/Gly/Leu), average 1.5 eV (P-Met).		[48]
5	Aβ	K_8_[P_2_NiW_17_O_61_], K_8_[P_2_CoW_17_O_61_]	Inhibition of Aβ fibrils formation via electrostatic attraction and His-chelating effect.	Aβ aggregation inhibition IC_50_: Ni-POM 5.67 ± 1.53 μM, Co-POM 14.73 ± 2.37 μM; BBB penetration: Brain W peak 0.0231 mg/kg (Ni-POM, 25 mg/kg iv); In vivo metabolism: Returns to initial concentration at 48 h (Ni-POM); Self-cytotoxicity: No significant toxicity (≤120 μM).		[15]
6	Aβ	K_8_[Co(H_2_O)_4_[HP_2_Mo_5_O_23_]_2_	POM blocks Aβ β-sheet folding via Co^2+^ coordination with nitrogen heterocycles and its oxygen-rich surface.			[49]
7	Aβ	{[CoL-(H_2_O)]_2_[CoL]_2_[HAs^V^Mo^VI^_6_O_40_]}	Target Aβ’s β-sheet via L group; inhibit/disaggregate Aβ via H-bonds and spatial embedding.	ROS scavenging: Reduced by 50% (Cu^2+^-Aβ induced); Cell viability: >40% (12 μM CAM); BBB penetration: Brain Mo peak ≈ 0.25 mg/kg (80 mg/kg iv); In vivo toxicity: No adverse effects (80 mg/kg).		[50]
8	Aβ	[MnMo_9_]-D-Phe, [MnMo_9_]-L-Phe	Bind to the α/β discordant segment of Aβ via hydrophobic interactions, π–π stacking, electrostatic interactions, and hydrogen bonds, disrupting its aggregation-related effects.	ROS scavenging: D-Phe-modified 106%; L-Phe-modified 116% (10 μM); BBB penetration: 1.98% (D-Phe-modified, 25 mg/kg iv); High-concentration toxicity: No significant toxicity (320 μM).		[35]
9	Aβ	(Me_4_N)_3_[PW_11_O_40_(SiC_3_H_6_NH_2_)_2_PtCl_2_] *	Pt^2+^ coordinates with Aβ_42_ amino acids and binds the HHQK cationic cluster via electrostatics, hydrogen bonding, van der Waals forces, and π–π stacking, inhibiting aggregation; its planar conformation disrupts fibrils and promotes degradation.	IC_50_: 0.62 μM; β-sheet reduction: 45.3 ± 0.9% → 41.4 ± 1.1% (8 μM)/41.2 ± 1.5% (16 μM); Fibril disassembly: 33–92% (10–100 µM) Cell viability (PC12): 49% → 67% (8 μM); In vivo (1.5 mg/mL): Passive avoidance latency 21.57 s → 76.74 s.		[51]
10	Aβ	(H_2_dap)_6_[Cd_4_Cl_2_(B-α-AsW_9_O_34_)_2_]	Surface O atoms form H-bonds with Aβ; Cd^2+^ coordinates His residues and competes with Zn^2+^/Cu^2+^, inhibiting misfolding and β-sheet aggregation.	ROS production decreased by 50% (in the Cu^2+^-β system, 20 µM); cell viability improved from <30% to >60%.		[52]
11	Aβ	POMD-TZ	Site-selectively modifies Aβ and binds its cationic region to inhibit Aβ aggregation.	BBB penetration: ~1.59% (brain W conc. 0.256 mg/kg, delayed by ~10 min)		[53]
12	Aβ	[(CH_3_)_2_NH_2_]_15_{α-P_2_W_15_Zr_3_(L-tartH)[αP_2_W_16_]} *, [(CH_3_)_2_NH_2_]_15_{α-P_2_W_15_Zr_3_(D-tartH) [α-P_2_W_16_]} *	Binds the HHQK cationic cluster of Aβ via electrostatic interactions; L/D-tartrate forms hydrogen bonds with Aβ, inhibiting aggregation; W-containing structure scavenges ROS via W^5+^/W^6+^ redox cycling.	Aβ aggregation inhibition: L-POM 43.90%, D-POM 26.45%; IC_50_: L-POM 17.38 μM, D-POM 2.63 μM; Binding constants: D-POM 1.07 × 10^6^ M^−1^, L-POM 5.40 × 10^5^ M^−1^ ROS scavenging: ~70% at 50 μM (both); PC12: Aβ-induced ROS 143%/viability 42%; Brain biodistribution: L-POM peak at 4 h, D-POM at 6 h.	Inhibit Aβ aggregation + scavenge ROS	[36]
13	Aβ	POM@P	The negatively charged surface of POM@P binds Aβ cationic clusters, increasing local peptide density, while the released POMs synergistically inhibit Aβ aggregation.	Aβ aggregation inhibition effect: ThT fluorescence inhibition rate over 65%; Cell protection effect: Cell viability increased to 82% (6 μM).		[54]
14	Aβ	AuNPs@POMD-pep	POMD binds Aβ via electrostatic and hydrogen-bond interactions, while the peptide segment binds Aβ through hydrophobic interactions, synergistically inhibiting aggregation and dissociating fibrils.	Aβ aggregation inhibition rate (40 nm): 47%; Aβ fibril dissociation rate (40 nm): 37%; Aβ-mediated peroxidase activity inhibition rate (1 nm): 63%; Cell survival rate (5 nm): >90%; IC_50_ ratio (vs. N-Ac-CLPFFD): 1/6.14 (6.14x lower); IC_50_ ratio (vs. AuNPs@POMD): 1/4.31 (4.31x lower).		[55]
15	Aβ	Peptide@Mo-POMs *	Peptide blocks Zn^2+^ sites on Aβ; Mo-POMs chelate Zn^2+^, synergistically inhibiting aggregation and disrupting protofibrils.	Apoptosis rate: 58.8% → 28.0%; Cell viability: 38.9% → >75%; ROS level: +107.9–38.6%.	Chelation of Zn^2+^ + Inhibition of Aβ Aggregation + Disassembly of Aβ Protofibrils	[37]
16	Aβ	CP-POM *	CP-POM inhibits Aβ_42_ aggregation and dissolves pre-aggregates through hydrogen bonding, hydrophobic interactions, and metal chelation.	Aβ42 fibril inhibition: ~45% (500 nM); Aβ42 fibril inhibition: 75% (100 nM); Aβ42 oligomer inhibition: 85% (100 nM); Preformed Aβ42 aggregate dissolution: 50% (100 nM); Neuronal viability improvement: ~64% (500 nM).	Inhibition of Aβ_42_ aggregation, dissolution of Aβ_42_ oligomers, metal chelation, and ROS scavenging	[56]
17	Aβ	AuNPs@POM@PEG	Inhibit Aβ aggregation via POM-Aβ binding.	Aβ inhibition: 75% (in vitro); Non-cytotoxic: <2.5 nM (neurovascular cells); BBB Pe: 3.47 vs. 3.07 × 10^−6^ cm/s.		[38]
18	Aβ	AuP	Aβ15-20 targeting → POMs/peptide inhibition → NIR photothermal Aβ fibril disassembly	BBB Penetration: 2.097 ± 0.337% (Brain Au accumulation); PC12 viability ↓ ~46% (Aβ 5μM).		[57]
19	Aβ	rPOMDs@MSNs@ copolymers *	Under NIR laser: generates local hyperthermia to disaggregate Aβ fibrils; releases rPOMDs to inhibit Aβ aggregation.	Aβ fibril disaggregation: ThT ↓ 32.7% (with NIR); Aβ aggregation inhibition: ThT ↓ 74.7%; turbidity 0.024; ROS scavenging: ↓ 52.8%; Cytoprotection: Cell viability ↑ 92.6% (Aβ + NIR).	Inhibit Aβ + Disaggregate preformed Aβfibrils + Scavenge ROS	[58]
20	Aβ	AuNPs@POMD-8pep *	Inhibits Aβ aggregation via electrostatic and sequence-specific binding, cleaves fibrils through histidine-mediated protease-like activity, scavenges ROS via redox-active SOD-like sites, and chelates Cu^2+^ to block metal-induced Aβ aggregation.	Protease specific activity: (8.80 ± 0.32) × 10^5^ U·mg^−1^; BBB penetration: Brain concentration peaks at 1 h post-administration.	Inhibit Aβ + Hydrolyze Aβ + chelate Cu^2+^	[59]
21	Aβ	CeONP@POMs *	POMD catalyzes Aβ peptide bond cleavage, electrostatic interactions inhibit/disaggregate Aβ aggregation, Ce^3+^/Ce^4+^ redox activity cooperatively scavenges ROS.	Intracellular ROS reduction rate: 68% (Aβ_40_-induced PC12 cells); BBB penetration efficiency: 4.4 ± 0.46% (in vitro), ~0.54% (in vivo).	Aβ cleavage + Aβ aggregation inhibition/disaggregation + ROS scavenging	[60]
22	S100A9	[N(CH_3_)_4_]_6_[Nb_10_O_28_], [N(CH_3_)_4_]_7_[TiNb_9_O_28_]	By electrostatic interactions with Lys-rich regions on the S100A9 surface, it induces local conformational changes that inhibit amyloid aggregation.	Nb10: S100A9 binding Kd: 2.86 ± 0.39 μM (intrinsic fluorescence), 1.17 ± 0.03 μM (ANS); TiNb9: S100A9 binding Kd: 2.48 ± 0.2 μM (intrinsic fluorescence), 0.45 ± 0.03 μM (ANS).		[61]
23	AchE, BchE	Na_10_[H_2_W_12_O_42_]	Inhibition of AChE/BChE via non-competitive mechanism.	AChE IC_50_: 2.30 ± 0.44 μM; BChE IC_50_: 1.56 ± 0.46 μM.		[41]
24	AchE, BchE	Na_16_[(O_3_POPO_3_)_4_W_12_O_36_]	Inhibition of AChE/BChE via electrostatic binding-altered active site.	AChE IC_50_: 3.51 ± 1.84 μM; BChE IC_50_: 0.18 ± 0.05 μM.		[41]
25	AchE, BchE	Na_16_[(O_3_PCH_2_PO_3_)_4_W_12_O_36_]	Inhibition of AChE/BChE via non-competitive mechanism.	AChE IC_50_: 5.04 ± 1.06 μM; BChE IC_50_: 0.18 ± 0.05 μM.		[41]
26	AchE, BchE	Na_6_[TeW_6_O_24_]	Inhibition of AChE/BChE via non-competitive mechanism.	AChE IC_50_: 0.31 ± 0.01 μM; BChE IC_50_: 0.46 ± 0.01 μM.		[41]
27	AchE	K_7_[Ti_2_PW_10_O_40_], K_6_H[SiV_3_W_9_O_40_]	Inhibition of AChE activity via inducing AChE conformation perturbation.	K_7_[Ti_2_PW_10_O_40_]: AChE inhibition IC_50_: 1.04 × 10^−6^ mol/L; K_6_H[SiV_3_W_9_O_40_]: AChE inhibition IC_50_: 4.80 × 10^−4^ mol/L.		[62]
28	AchE	H_4_[SiW_12_O_40_], H_3_[PW_12_O_40_]	Through electrostatic and hydrogen-bond interactions with the β-allosteric site (β-AS) of AChE, enzymatic activity is inhibited, preventing the α-helix-to-β-sheet transition of the AChE_586–599_ region.	WSiA: AChE inhibition IC_50_: 72.3 ± 0.2 nM; logP: −0.47; Hill coefficient: 0.93 ± 0.09; Cytostasis: 2.62%~11.24% (1 × 10^−6^~1 × 10^−4^ M) WPA: AChE inhibition IC_50_: 1230.0 ± 10.0 nM; logP: −0.29; Hill coefficient: 1.23 ± 0.17; Cytostasis: 7.87%~11.61% (1 × 10^−6^~1 × 10^−6^ M)		[63]

* POMs marked with * demonstrate multi-target effects, highlighting their potential for cross-pathway intervention.

## Data Availability

The authors permit the sharing of all the data. The data presented in this study are available on request from the corresponding authors.
